# Activity Tracking Using Ear-Level Accelerometers

**DOI:** 10.3389/fdgth.2021.724714

**Published:** 2021-09-17

**Authors:** Martin A. Skoglund, Giovanni Balzi, Emil Lindegaard Jensen, Tanveer A. Bhuiyan, Sergi Rotger-Griful

**Affiliations:** ^1^Division of Automatic Control, Department of Electrical Engineering, The Institute of Technology, Linköping University, Linkoping, Sweden; ^2^Eriksholm Research Centre, Oticon A/S, Snekkersten, Denmark; ^3^Department of Electrical Engineering, Technical University of Denmark, Ørsteds Plads, Lyngby, Denmark; ^4^Oticon A/S, Smorum, Denmark

**Keywords:** activity tracking, accelerometer, classification, machine learning, supervised learning, hearing aids, hearing healthcare

## Abstract

**Introduction:** By means of adding more sensor technology, modern hearing aids (HAs) strive to become better, more personalized, and self-adaptive devices that can handle environmental changes and cope with the day-to-day fitness of the users. The latest HA technology available in the market already combines sound analysis with motion activity classification based on accelerometers to adjust settings. While there is a lot of research in activity tracking using accelerometers in sports applications and consumer electronics, there is not yet much in hearing research.

**Objective:** This study investigates the feasibility of activity tracking with ear-level accelerometers and how it compares to waist-mounted accelerometers, which is a more common measurement location.

**Method:** The activity classification methods in this study are based on supervised learning. The experimental set up consisted of 21 subjects, equipped with two XSens MTw Awinda at ear-level and one at waist-level, performing nine different activities.

**Results:** The highest accuracy on our experimental data as obtained with the combination of Bagging and Classification tree techniques. The total accuracy over all activities and users was 84% (ear-level), 90% (waist-level), and 91% (ear-level + waist-level). Most prominently, the classes, namely, standing, jogging, laying (on one side), laying (face-down), and walking all have an accuracy of above 90%. Furthermore, estimated ear-level step-detection accuracy was 95% in walking and 90% in jogging.

**Conclusion:** It is demonstrated that several activities can be classified, using ear-level accelerometers, with an accuracy that is on par with waist-level. It is indicated that step-detection accuracy is comparable to a high-performance wrist device. These findings are encouraging for the development of activity applications in hearing healthcare.

## 1. Introduction

A strong trend in modern hearing aid (HA) development and research is the inclusion of more sensing technologies. The driver behind this is the wish for better, more personalized, and self-adaptive (1–3) devices that can handle environmental changes (4–7) and cope with day-to-day fitness of the users. Current HAs usually try to analyze the soundscape and adjust the settings according to a formula. However, recent HAs have advanced further by combining sound analysis with motion activity classification based on accelerometers to adjust settings with the aim of a better user experience. A few other possible uses of accelerometers in HAs are as follows: fall detection (8) to alert caretakers; tap detection for user interfacing (9); and health monitoring based on physical activity (10). The backbone of the above-mentioned applications is accurate and robust activity tracking that can determine and distinguish between several relevant activities, e.g., standing, sitting, walking, running, and more. While a lot of research in activity tracking and classification using accelerometers has been in sports applications (11–14) and general consumer electronics (15–17), such as smart-watches and cell phones, the hearing research body is small. The results in this contribution is based on the work in Balzi (18). The background to this study is that accelerometers are, or are to appear, in hearing devices and that it is of fundamental interest to investigate their usefulness in the activity tracking. The key objective of this study is to investigate the feasibility of activity tracking with ear-level accelerometers and how it compares to waist-mounted accelerometers, which is the more common measurement location in sports and healthcare. The activity classification method is based on supervised learning on experimental data from 21 subjects. The scope of the investigation is limited to 21, normal hearing, healthy subjects, and 9 activities.

## 2. Method

This section outlines the relevant details of the activity tracking methodology based on accelerometer data and machine learning.

### 2.1. Accelerometer Measurements

It is assumed that the sensors are mounted rigidly onto the users and that any kind of mounting play is negligible. It is further assumed that sensor axes are orthogonal, that the sensitivities are known and linear in the working span, and that sensor biases are negligible. The assumed inertial (fixed) coordinate frame, with axes (*XYZ*), is a local, right-handed, Euclidean frame with the *Z*-axis parallel to the local gravity vector. The data from a tri-axial accelerometer are then


(1) $$\begin{array}{l} a=R\left(a^{i}-g\right)+e, \end{array}$$

where $a={\left[a_{x},a_{y},a_{z}\right]}^{T}$ is the body referenced measurement for the sensor axes (*xyz*), *R* is a rotation matrix relating the orientation of the inertial frame and the body frame, $a^{i}={\left[a_{X},a_{Y},a_{Z}\right]}^{T}$ is the acceleration in an inertial frame, the local gravity vector *g* ≈ [0, 0, 9.81]^*T*^*m*/*s*^2^ is assumed constant, and the noise, *e*, is assumed Gaussian distributed with the same standard deviation (SD), σ_*e*_, in each axis, $e\sim N\left(0,\sigma_{e}I\right)$. The measured forces can be divided in to static forces, such as, constant acceleration and gravity, and the dynamic forces that are due to motion of changing rate, e.g., nodding and shaking. Note that even in the ideal case without noise, it is not possible to solve (Equation 1) for *a*^*i*^ and other data, e.g., a magnetometer or a high-grade gyroscope is needed to resolve the rotation *R*, see Titterton et al. (19) for details. In most situations, the human body accelerations are small compared to gravity, and it is, therefore, possible to estimate the inclination, i.e., each sensor axis angle with respect to the gravity vector, which is related to the orientation in roll and pitch.

### 2.2. Accelerometer Features

In machine learning, feature extraction is pre-processing of data with the intention of increasing the overall performance of classifiers. The underlying idea is that certain transformations can yield more information, higher independence, and give larger margins for class separability. Feature selection is very much application dependent and usually require domain knowledge, though computationally expensive automated methods exist, [see, e.g., (20)] for an overview. The selected features described below are inspired by the work of Masse et al. (21), Gjoreski et al. (22), and Hua et al. (23) and have been adapted with this application in mind. The features are defined from 13 metrics of which 10 are applied to each axis, resulting in a total of 33 features as described below.

#### 2.2.1. Tilt Angles

The tilt angles, sometimes referred to as inclination, are defined as


(2) $$\begin{array}{l} \phi_{k}=\arccos\left(\frac{a_{k}}{r}\right),\;k=\left\{x,y,z\right\}, \end{array}$$

where


(3) $$\begin{array}{l} r=\sqrt{a_{x}^{2}+a_{y}^{2}+a_{z}^{2}} \end{array}$$

and is indicative of each axis angle with respect to the local gravity vector. Errors in the tilt angles arise from the presence of motion and noise.

#### 2.2.2. Acceleration Vector Change

The acceleration vector change (AVC) is a motion-sensitive metric defined by the mean of the absolute value of the differences in the acceleration vector length (Equation 3), and the mean is calculated in a window with size *M* as


(4) $$\begin{array}{l} AVC=\frac{1}{M}\overset{M}{\underset{i=1}{\sum }}\frac{\left|r_{i+1}-r_{i}\right|}{T_{s}} \end{array}$$

where *i* denotes samples the *i*^th^ sample at a given, fixed, sampling frequency *f*_*s*_ with the sampling interval *T*_*s*_ = 1/*f*_*s*_.

#### 2.2.3. Signal Magnitude Area

The signal magnitude area (SMA) is defined over a window with size *M* as


(5) $$\begin{array}{l} SMA=\frac{1}{M}\overset{M}{\underset{i=1}{\sum }}|a_{x_{i}}|+|a_{y_{i}}|+|a_{z_{i}}| \end{array}$$

and it is a measure of the magnitude.

#### 2.2.4. Mean and SD

The mean (Equation 6) and SD (Equation 7) are computed for each axis over a window with size *M* as


(6) $$\begin{array}{l} \mu_{k}=\frac{1}{M}\overset{M}{\underset{i=1}{\sum }}a_{k_{i}},\;k=\left\{x,y,z\right\}, \end{array}$$

(7) $$\begin{array}{l} \sigma_{k}=\sqrt{\frac{1}{M}\overset{M}{\underset{i=1}{\sum }}{\left(a_{k_{i}}-\mu_{k}\right)}^{2}},\;k=\left\{x,y,z\right\}. \end{array}$$

#### 2.2.5. Root Mean Square

Similar to the SD (Equation 7), the root mean square (RMS) is computed for each axis over a window of size *M* as


(8) $$\begin{array}{l} RMS_{k}=\sqrt{\frac{1}{M}\overset{M}{\underset{i=1}{\sum }}a_{k_{i}}^{2}},\;k=\left\{x,y,z\right\}. \end{array}$$

#### 2.2.6. Minimum and Maximum

Minimum (MIN) and maximum (MAX) values per axis over a window with size *M* are defined by


(9) $$\begin{array}{l} MIN_{k}=\min{\left\{a_{k_{i}}\right\}}_{i=1}^{M},\;k=\left\{x,y,z\right\} \end{array}$$

and


(10) $$\begin{array}{l} MAX_{k}=\max{\left\{a_{k_{i}}\right\}}_{i=1}^{M},\;k=\left\{x,y,z\right\} \end{array}$$

respectively.

#### 2.2.7. Median

The median is the center value of a size-ordered sample, and it is not skewed by large or small values as the mean is. The median, can e.g., be used to detect burst noise outliers in data and is computed for each axis over a window of size *M* as


(11) $$\begin{array}{l} MEDIAN_{k}=\mathrm{median}\left({\left\{a_{k_{i}}\right\}}_{i=1}^{M}\right)\;k=\left\{x,y,z\right\}. \end{array}$$

#### 2.2.8. Median Absolute Deviation

The median absolute deviation (MAD) is a measure of sample variability around the median. The MAD is computed for each axis over a window of size *M* as


(12) $$\begin{array}{l} MAD_{k}=\mathrm{median}\left({\left\{a_{k_{i}}-MEDIAN_{k}\right\}}_{i=1}^{M}\right),\;k=\left\{x,y,z\right\} \end{array}$$

where the *MEDIAN* from Equation (11) is used.

#### 2.2.9. Skewness

The skewness (SKW) is the third standardized moment of a sample and is a measure of the asymmetry of a distribution about the mean. Using the previously defined μ_*k*_ (Equation 6) and σ_*k*_ (Equation 7), the sample SKW is computed for each axis over a window of size *M* as


(13) $$\begin{array}{l} SKW_{k}=\frac{\frac{1}{M}\overset{M}{\underset{i=1}{\sum }}{\left(a_{k_{i}}-\mu_{k}\right)}^{3}}{\sigma_{k}^{3}},\;k=\left\{x,y,z\right\}. \end{array}$$

Note that other approximations for sample skewness are possible (24).

#### 2.2.10. Counts per Second

Counts per second (counts/s) is a widely used measure in the activity tracking. The computation of counts/s is proprietary of ActiGraph LLC and is usually carried out with ActiGraph accelerometer devices. However, Crouter et al. (25) details on how to derive this measure on a standard accelerometer and for the work here our Matlab implementation is based on Brønd et al. (26).

### 2.3. Machine Learning

The activity classification is based on a supervised learning to train the classifier. Three classification methods are considered in this study, K-nearest-neighbor (KNN), linear-discriminant-analysis (LDA), and decision tree (DT). Further improvement of the classification can be obtained using ensemble learning methods such as Boosting and Bootstrap aggregation (Bagging), and variations thereof.

#### 2.3.1. Classifiers

In supervised learning, a set of training instances with corresponding class labels is given, and a classifier is trained and used to predict the class of an unseen instance, [see, e.g., (27)] for details. The *N* samples of training data *x* and class labels *y* were ordered in pairs {(*x*_1_, *y*_1_), …, (*x*_*N*_, *y*_*N*_)} such that the *i*-th feature vector $x_{i}\in {\mathbb{R}}^{p}$ corresponds to the binary class label vector $y_{i}\in {\mathbb{Z}}_{2}^{c}$. For the case with a single tri-axial accelerometer and the features described in section 2.2, the feature vector dimension, *p*, is 33 per sample while the class label vector dimension *c* is 9.

The three well-known, but rather different, supervised classifiers are considered, and the choice to use these was based on the availability of good implementations. The classifiers are as follows:

- The KNN classifier (28, 29) is here based on the Euclidean distance between the test- and training samples. However, other distance measures can be used.
- The LDA, or Fisher's discriminant (30), is a statistical method to find linear combinations in the feature space to separate the classes, and it is carried out by solving a generalized eigenvalue problem.
- DT has a flow chart-like structure where the root corresponds to feature inputs, the branches of the descending test-nodes represents the outcome of the test, and each leaf-node represents a class label, [see, e.g., (31)].

#### 2.3.2. Ensemble Training

Ensemble training is used to increase the predictive classification (or regression) performance by learning a combination of several classifiers. The two main categories used here are Bagging (31) and Boosting (32) with a few selected variations. In Bagging, the classifiers are trained in parallel on randomly sampled training data while in Boosting the training is carried out sequentially as the classifiers and data are weighted according to their importance. The first, and most well-known, Boosting algorithm is called AdaBoost (short for Adaptive Boosting) and was originally formulated in Freund and Schapire (33).

## 3. Experiment

Experimental data were collected from 21 voluntary subjects performing a series of tasks representative of the stipulated activities. A total of three tri-axial accelerometers were used. Experimental data were collected jointly in the projects (18, 34).

### 3.1. Subjects

Prior to the experiments, the subjects were informed about the experiment procedure and the use of data before deciding on their participation with oral consent. Data were stored and labeled anonymously. The 21 subjects had an age range between 24 and 60 years old, N(32.6,9.6), both women and men with a height bracket of 1.55−195 *m*, N(1.78,0.091). None of the subjects had any reported health issues. The subjects did not receive any compensation.

### 3.2. Data

**Figure 1:**
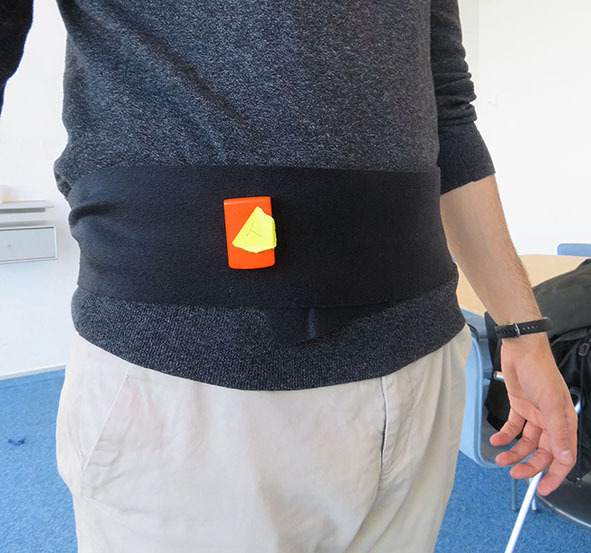
Ear-level accelerometer placement used in the experiment.

**Figure 2:**
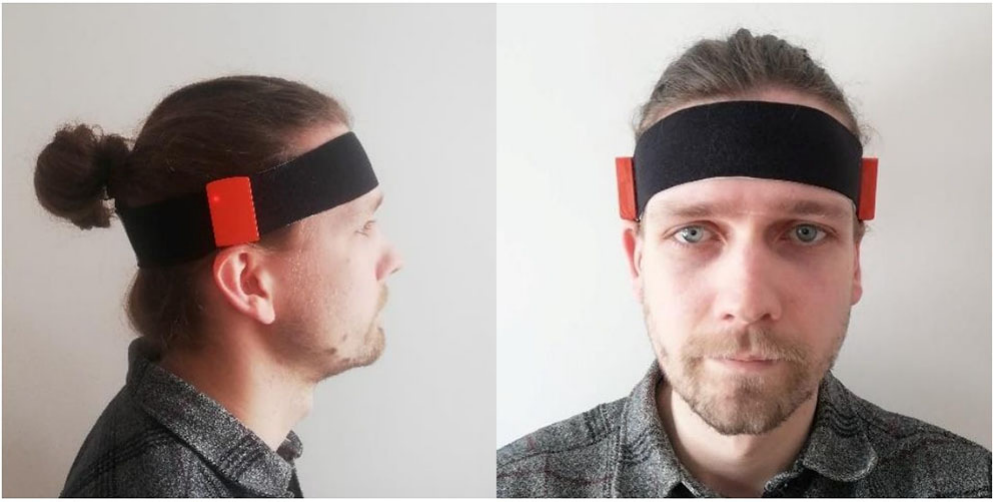
Waist-level accelerometer placement used in the experiment.

For all subjects, two of the accelerometers were placed on each side of the head at ear-level, see Figure 1, to mimic HA sensors and the third accelerometer was placed at waist-level using an elastic strap, see Figure 2, as it is a more common region for activity measurements and it is also representative of an in-pocket smartphone.

The accelerometers are XSens MTw Awinda produced by XSens Technologies B.V. and are battery powered, wireless devices, inertial sensors containing tri-axial accelerometers and gyroscopes and also tri-axial magnetometers enabling accurate orientation estimation when the device is stationary. The data are collected wirelessly using the MT Manager software by XSens on a PC laptop running Microsoft Windows 10 at a sampling rate, *f*_*s*_, of 100 *Hz* that was decided to be fast enough for the intended activities. Data are manually labeled based on visual inspection during the experiment to match the activities in section 3.4.

### 3.3. Task

The experiment was carried out in a room with a soft carpet at Oticon main offices, Smørum, Denmark. For tasks involving lying down and falling, a mattress was used. Each of the 21 subjects from whom the data have been gathered was asked to perform 6 different tasks while wearing all three accelerometers, and between each task all the data from the accelerometers were saved and anonymously cataloged. Except for the accelerometer data, from each subject, only gender, age, and height were collected. To every subject, the same specific information about which actions to be carried out was given by reading out loud from a manuscript, and no restrictions were communicated regarding how to carry out the various exercises with the intention of increasing the possibility of movement variability in the activities. With more in-class variation used for training, the classifier is less prone to over-fitting at the expense of higher probability of between-class overlap. The mean test duration was about 22 min, including pauses, and generated about 13–14 min of data per subject.

### 3.4. Physical Activities

The choice of activities to track is a trade-off between how clearly activities can be discerned from each other, the likelihood of activities being present in the daily routines of the subjects, and the intended use of activity tracking. A typical scenario, not addressed here, is that HA users often remove the HAs when lying down to rest. Hence, for a HA applications, in-ear detection, e.g., using accelerometers could be useful. The fidelity of activity categories is chosen as either resting or moving, and no intensity or within-class variation is considered.

The resting activities are as follows:

Act1 :**Standing** in a still position.
Act2 :**Sitting**
Act3 :**Lying face-up (LFU)**
Act4 :**Lying face-down (LFD)**
Act5 :**Lying side (LS)** on either left or right side

and the moving activities are as follows:

Act6 :**Walking, on the floor**
Act7 :**Jogging, moderate pace in circles/square**
Act8 :**Falling** on whichever side, some subjects could not simulate a perfect falling motion and, therefore, have been asked to perform a fast transition from standing to Lying down, at top of their capability.
Act9 :**Transitioning (TRN)** all instances not being any of the other activities, e.g., going from one activity to another.

Note that there is no specific class for head motion as it was predicted being difficult to correctly label and that there is already significant head motion within all the moving activities 3.4 to 3.4. With e.g., a waist-level accelerometer, it may be possible to separate head motions from general body motions, but it is beyond this study.

## 4. Results

As an initial step, 200 min of the dataset in Anguita et al. (35) was used on all combinations of classfiers and ensemble training methods described in Section 2.3. The data are open, pre-labeled, in-pocket cell phone, and it has six activity classes: walking; walking stairs up; walking stairs down; sitting; standing; and laying. For the use here, all the walking classes were considered the same. The best predictive classification accuracy was obtained using the DT and Bagging, and it was furthermore also the case for our experimental data. Consequently, all following results are obtained using DT and Bagging.

### 4.1. Pre-processing

The data from the 21 subjects were randomly partitioned in to two groups with all activities present in both groups and with 70% used for training and 30% used for testing (validation). Training with cross-validation is carried out for as many times as there are trees, i.e., 100–500 depending on the setup. The tree depth is 480 with 13,121 nodes. Features are computed for each sample at 100 *Hz*with the window size to one sample, *M* = 1 for the features AVC and SMA, while *M* = *f*_*s*_ = 100, centered at the current sample, for the other applicable features. All results are obtained using the Machine Learning Toolbox in Matlab 2021b and with dependencies to the Optimization Toolbox for certain classifiers.

### 4.2. Classification

The main performance target here is the accuracy of predicted class labels in data not used for training as it is a common measure in supervised learning, [see, e.g., (11, 12, 14, 36, 37)]. The accuracy is defined as the number of correctly classified labels divided by the total number of labels and simply states how much of the data not used for training that is correctly classified. In Table 1, the classification result using both ear-level accelerometers is illustrated based on 500 Decision trees and Bagging trained with a learning rate of 0.1 and showing an overall predicted accuracy of 84.4%. Most prominently, the classes, namely, standing 3.4, jogging 3.4, laying side 3.4, lying face-down 3.4, and, walking 3.4, all have an accuracy of above 90%. The lowest scoring activities are as follows: falling 3.4, sitting 3.4, and transitioning 3.4. The falling activity is often confused with transitioning, which, in turn, generally is confounded with all other activities. The overall accuracy is more than 90% without the sitting activity.

**Table 1:** Confusion matrix of the predicted accuracy with Bagging and Decision Tree using both ear-level accelerometers.

	**LFD**	**LFU**	**Falling**	**Jogging**	**LS**	**Sitting**	**Standing**	**TRN**	**Walking**	**C**	**NC**
LFD	6,294				1,221			93		82.73	17.27
LFU	1,108	22,754			55			195		94.37	5.63
Falling		87	364		3		16	323	27	44.39	55.61
Jogging			266	43,928			231	932	1,807	93,14	6.86
LS					7,311			26		99.65	0.35
Sitting						2,347	33,213	242	13	6.55	93.45
Standing	8					2,108	1,42,889	1,804	1,496	96.35	3.65
TRN	357	1,082	642	107	116	10	8,135	27,138	4,399	64.64	35.36
Walking			43	28		2	3,505	11,865	1,58,433	91.12	8.88
C	81.04	95.11	27.68	99.69	83.98	52.54	76.01	63.68	95.34		
NC	18.96	4.89	72.32	0.31	16.02	47.46	23.99	36.32	4.66		

*The row-normalized row summary on the right displays the percentages of correctly and incorrectly classified observations for each true class, while the column-normalized column summary below the matrix displays the percentages of correctly (C) and incorrectly (NC) classified observations for each predicted class*.

### 4.3. Feature Evaluation

From a computational perspective, it is good to minimize the number of features needed and, therefore, the relative importance of features for each activity is analyzed using 100 Decision trees and Bagging, with a learning rate of 0.1. In Table 2, the total accuracy per feature (or pairs in some cases) for each activity is considered where accelerations only on the top row are considered that base model and the contribution of each additional feature and activity are below. Notably, the tilt angles have only a marginal positive effect on the sitting activity and mostly negative effect on all other activities. Other features with little importance are RMS and counts/s. In Table 3, the increase (or regression) per feature and activity, compared to the base level accelertions only, is shown. The overall most important features are: mean, SD, MIN, MAX, median, and MAD. Furthermore, in Table 4 a summary of the best and worst activity per feature is shown and, as expected, the best ranking features are important for several activities.

**Table 2:** Total accuracy per feature (or pairs of features) per activity compared to just using accelerations (ACC) only.

	**OA**	**LFD**	**LFU**	**Falling**	**Jogging**	**LS**	**Sitting**	**Standing**	**TRN**	**Walking**
ACC only	72.17	93.70	85.40	13.70	58.70	71.70	5.80	91.90	39.40	78.10
Tilt Angles	71.67	81.80	79.50	14.00	58.80	69.10	6.30	91.80	39.50	78.10
AVC	75.50	95.00	94.30	16.10	70.30	76.00	5.70	93.10	40.20	81.60
SMA	75.47	95.20	94.20	17.20	70.20	74.90	5.90	93.00	40.20	81.60
Mean + SD	82.71	98.80	94.40	36.00	90.00	99.70	10.70	94.10	60.30	88.40
RMS	72.53	93.70	83.40	14.40	58.70	97.20	5.80	92.00	39.50	78.10
MAX + MIN	82.50	90.30	94.40	33.00	89.50	99.20	10.50	94.10	57.90	89.00
Median + MAD	81.76	99.10	94.60	28.90	89.90	98.80	10.90	94.40	54.00	87.10
Skewness	76.17	95.00	88.70	13.30	52.10	87.30	4.60	95.80	47.40	84.90
Counts/Sec	71.73	78.60	80.80	14.40	58.70	74.30	5.80	91.90	39.50	78.00

*The overall accuracy (OA) in the left-most column is the effect when adding each feature*.

**Table 3:** Accuracy improvement per feature and activity.

	**OA**	**LFD**	**LFU**	**Falling**	**Jogging**	**LS**	**Sitting**	**Standing**	**TRN**	**Walking**
ACC only	0.00	0.00	0.00	0.00	0.00	0.00	0.00	0.00	0.00	0.00
Tilt Angles	−0.50	−11.90	−5.90	0.30	0.10	−2.60	0.50	−0.10	0.10	0.00
AVC	3.33	1.30	8.90	2.40	11.60	4.30	−0.10	1.20	0.80	3.50
SMA	3.30	1.50	8.80	3.50	11.50	3,20	0.10	1.10	0.80	3.50
Mean + SD	10.54	5.10	9.00	22.30	31.30	28.00	4.90	2.20	20.90	10.30
RMS	0.36	0.00	−2.00	0.70	0.00	25.50	0.00	0.10	0.10	0.00
MAX + MIN	10.33	−3.40	9.00	19.30	30.80	27.50	4.70	2.20	18.50	10.90
Median + MAD	9.59	5.40	9.20	15.20	31.20	27.10	5.10	2.50	14.60	9.00
Skewness	4.00	1.30	3.30	−0.40	−6.60	15.60	−1.20	3.90	8.00	6.80
Counts/s	−0.44	−15.10	−4.60	0.70	0.00	2.60	0.00	0.00	0.10	−0.10

*Same setup as in Table 2 but corrected for baseline accuracy obtained by accelerations only*.

**Table 4:** Best- and worst-case per feature accuracy using the features listed in the left column based on Table 3.

**Feature**	**Best activity**	**Worst activity**
ACC only	X	X
Tilt Angles	X	Lying face-down
AVC	Jogging, Lying face-up	X
SMA	Jogging, Lying face-up	X
Mean + SD	Jogging, Lying face-up, Falling, Transitioning	X
RMS	Lying side	Lying face-up
MAX + MIN	Jogging, Transitioning, Falling, Lying side	Lying face-down
Median + MAD	Jogging, Lying side, Transitioning, Falling	X
Skewness	Lying side	Jogging
Counts/s	Lying side	Lying face-down

*Cells marked with X means that there is no improvement or regression in using that feature*.

### 4.4. Sensor Combinations

One of the main motivations of this study is to compare the feasibility of ear-level activity tracking compared to waist-level sensoring. For all sensor combinations and data types the same features and training were computed to get the comparable classification results. In Table 5, a confusion matrix shows the results of using only the waist-level accelerometer, giving an overall accuracy of 89.6% In Table 6, a confusion matrix showing the result from two ear-level accelerometers and the waist-level accelerometer is used together with 500 Decision trees, giving an overall accuracy of 91.6%. One of the reasons for the improvement is that the addition of the waist-level accelerometer makes the sitting activity easier to distinguish with 94.4% correct compared to 52.5% when using only ear-level accelerometers and this performance increase also shows on the standing activity as the previously discussed confusion is decreased. In Table 7, the overall accuracy using the various combinations of sensors is shown; for instance, 25 *Hz* accelerometer data from a wrist-worn Garmin Vivosmart 4 are used with only a 56.3% accuracy. In Table 7, 100 Decision trees compared to the previous 500 were chosen to save computations and the accuracy decrease is negligible. The gyroscope and orientation data are obtained from the XSens devices. Note that the orientation data are adapted for stationary orientations and, therefore, of low-pass characteristics and potentially not well suited for all aspects of this application. It can be noted that the waist-level accelerometer alone is rather efficient and, as noted before, the combination with the ear-level accelerometers gives even better performance.

**Table 5:** Confusion matrix using Bagging and Decision Tree using only the waist accelerometer.

	**LFD**	**LFU**	**Falling**	**Jogging**	**LS**	**Sitting**	**Standing**	**TRN**	**Walking**	**C**	**NC**
LFD	6,993							615		91.92	8.08
LFU	1,181	21,752						1,206		90.11	9.89
Falling	18	66	413		22		26	252	23	50.37	49.63
Jogging			23	44,008			360	306	2,467	93.31	6.69
LS	1,471				5664			202		77.20	22.80
Sitting						24,032	9,027	2,744	12	67.10	32.90
Standing			6	5		6,756	1,38,157	1,833	1,548	93.16	6.84
TRN	543	1,065	579	98	64	937	6,211	28,777	3,802	68.39	31.61
Walking			17	168		10	3,696	2,690	1,67,295	96.22	3.78
C	68.52	95.06	39.79	99.39	98.50	75.73	87.73	74.50	95.52		
NC	31.48	4.94	60.21	0.61	1.50	24.27	12.27	25.50	4.48		

*The row-normalized row summary on the right displays the percentages of correctly and incorrectly classified observations for each true class, while the column-normalized column summary below the matrix displays the percentages of correctly (C) and incorrectly (NC) classified observations for each predicted class*.

**Table 6:** Confusion matrix using Bagging and Decision Tree using both ear-level accelerometers, and the waist accelerometer.

	**LFD**	**LFU**	**Falling**	**Jogging**	**LS**	**Sitting**	**Standing**	**TRN**	**Walking**	**C**	**NC**
LFD	6,274				1,221			113		82.47	17.53
LFU	1,156	22,769			10			177		94.43	5.57
Falling		86	374		8		20	328	4	45.61	54.39
Jogging			91	44,120			245	632	2,076	93.55	6.45
LS					7,324			13		99.82	0.18
Sitting						24,202	10,175	1,436	2	67.58	32.42
Standing				6		844	1,44,503	1,369	1,556	97.45	2.55
TRN	254	1,010	533	78	112	591	5,947	30,698	2,493	73.59	26.41
Walking			33	47			3,339	4,563	1,65,894	95.41	4.59
C	81.65	95.41	36.28	99.70	84.43	94.40	87.99	78.05	96.44		
NC	18.35	4.59	63.72	0.30	15.57	5.60	12.01	21.95	3.56		

*The row-normalized row summary on the right displays the percentages of correctly and incorrectly classified observations for each true class, while the column-normalized column summary below the matrix displays the percentages of correctly (C) and incorrectly (NC) classified observations for each predicted class*.

**Table 7:** Accuracy with different sensor combinations using Bagging and 100 Decision trees.

	**L &**	**L, R**		**L R**		**L, R**		**L, R, &**	**L, R, &**	
**L**	**R**	**& W**	**W**	**ACC only**	**GYR**	**& GYR**	**ORI**	**ORI**	**Garmin**	**Garmin**
83,96	84,34	91,57	89,62	74,48	70,93	84,99	47,18	84,76	81,56	56,25

*Left (L), Right (R), and Waist (W) are short for the respective accelerometer placements. GYR is short for Gyroscope, and ORI is short for Orientation*.

### 4.5. Step Detection

Another concrete measure that can be useful for activity tracking is step detection, which was also analyzed in Acker (10). For the walking and jogging activities, ear-level step detection was computed based on Bai et al. (38) and Abadleh etal. (39) using the AVC feature resulting in 95% and a 90% accuracy, respectively, using both ear-level accelerometers. This can be compared with the highly optimized Garmin Forerunner 35 giving a 99% (walking) and 95% (jogging) accuracy, respectively.

## 5. Discussion

The main objective of this study is to compare ear- and waist-level activity tracking performance. Therefore, it is not fundamental to have features and classifiers that could outperform the works of others and such a comparison is beyond this study. While not directly comparing to other methods, the overall ear-level activity classification results are encouraging in the proposed setup.

As noted, it is difficult to separate falling and transitioning with ear-level data only. Possible explanations are that the selected features are not sensitive enough to distinguish between falling and transitioning and that transitioning is too general [or complex as Dernbach et al. (11)] and can, for instance, be confounded with general head movements. More controlled falling experiments, such as, Burwinkel and Xu (8) could provide useful insights. The sitting activity was confounded with the standing activity as they are typically rather similar, and the only potential differences at ear-level between the two may be in postural sway that should be clearer for standing subjects. This difficulty was also found in Parkka et al. (40), and an accelerometer below the waist is particularly useful here. At waist-level it is easier to distinguish sitting and standing and it is possibly explained by the change in the tilt angles for seated subjects.

Designing features sensitive to particular classes is an engineering task requiring expertise. As noted in section 4.3, some features are not that well-suited for any of the activities and would benefit from further tuning, such as, other pre-processing and different window sizes, or should otherwise be omitted. A feature that is sensitive to postural sway (low-frequency component) could potentially support separating standing and sitting at ear-level.

Sensor combinations can improve the results, [see, e.g., (14, 40)], and here the combination of ear- and waist-level data is the overall best. On another positive note, the accuracy difference between one and two ear-level devices is small and this is a good news for HA applications, as single-sided hearing compensation is common. The wrist data, here from the Garmin device, may be difficult in general as arms may do many types of motions not specifically relating to the activities.

The use of gyroscope data is common, [see, e.g., (41–43)], and was expected to improve the results in general. However, all sensor types were processed in the same fashion as accelerometer data with the same features and are a possible explanation of the poor performance of many of the additional data types in Table 7.

Wrist data were poor for activity tracking in the setup here, but the output of the proprietary algorithms on these types of devices suggests that a lot more can be achieved on ear-level devices too. The step detection is almost on par with the commercial Garmin device, for the short durations considered here, and these typically utilize additional sensors, e.g., magnetometer and gyroscope, and their algorithms can be considered state-of-the-art.

## 6. Conclusions

We investigated the feasibility of ear-level accelerometers for activity tracking in comparison to waist-level accelerometers. Many activities can be classified with an accuracy that is on par with a waist-level accelerometer, and this is particularly encouraging for the development of activity applications in hearing healthcare. Furthermore, we indicate that step-detection accuracy is comparable to a high-performance wrist device. It is also shown that higher predictive performance can be obtained when combining ear- and waist-level accelerometer data, and this could potentially assist in isolating head motion from full body activities, opening for a higher granularity of activity classes.

Noteworthy limitations in this study were as follows: the modest number of test subjects (21); the number of activities (9) per test subject; that the manual data labeling may have errors; and efforts spent on feature design and learning methods. Also, data with a more control and a clearer reference, e.g., a motion capture system, could provide valuable insight at the expense of more costly and complex experiments.

Future directions should consider further feature design with, e.g., multi-tapers and various transforms. The design should start with time-frequency analysis of activities for guidance. Experiments on a larger, more diverse population, with additional knowledge on head motion/orientation throughout, can open up for higher performance and other activity classes. The classification methods could be further improved considering the state-of-the-art in Deep Learning as initially explored by Ronao and Cho (37) and Hammerla et al. (44).

## Data Availability Statement

The raw data supporting the conclusions of this article will be made available by the authors, without undue reservation.

## Ethics Statement

Ethical review and approval was not required for the study on human participants in accordance with the local legislation and institutional requirements. Written informed consent for participation was not required for this study in accordance with the national legislation and the institutional requirements.

## Author Contributions

The majority of manuscript preparation was carried out by MS with assistance from all authors. GB generated results. GB, TB, and MS developed the methodology. GB and EJ carried out the experiments and data collection.

## Funding

This work was financially supported by the Swedish Research Council (Vetenskapsrådet, VR 2017-06092 Mekanismer och behandling vid åldersrelaterad hörselnedsättning).

## Conflict of Interest

MS, TB, and SR-G are employed by Oticon A/S. The remaining authors declare that the research was conducted in the absence of any commercial or financial relationships that could be construed as a potential conflict of interest.

## Publisher's Note

All claims expressed in this article are solely those of the authors and do not necessarily represent those of their affiliated organizations, or those of the publisher, the editors and the reviewers. Any product that may be evaluated in this article, or claim that may be made by its manufacturer, is not guaranteed or endorsed by the publisher.
